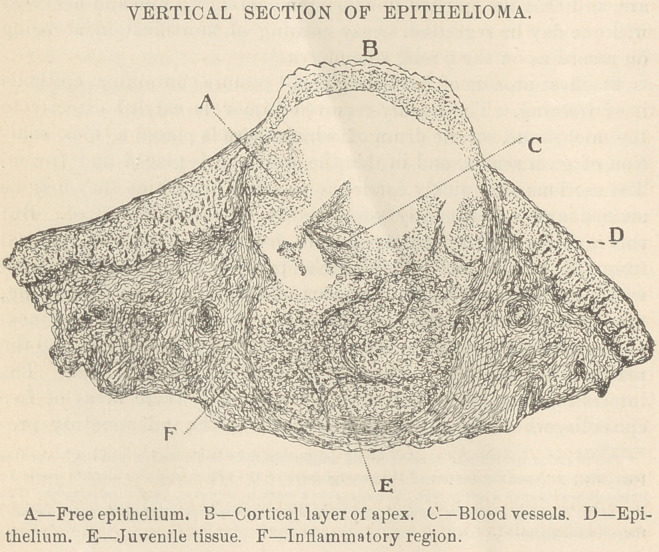# Epithelioma

**Published:** 1885-05

**Authors:** A. M. Ross

**Affiliations:** Chicopee, Mass.


### EPITHELIOMA.

BY DR. A. M. ROSS, CHICOPEE, MASS.

A gentleman of about forty-five years of age recently called my attention to a growth in the mucous membrane of his cheek, which appeared to be about the size of half a large pea. The body of it was smooth, with the margins well defined. The color was considerably darker than the surrounding tissues, except at the apex, where it was a dirty yellow. When his mouth was closed and his features were in repose, the tumor was opposite an opening in the lower dental arch, caused by the loss of a first bicuspid. The cuspid and second bicuspid on either side of this space were considerably worn by mastication. The teeth being of a very dense structure, the angles caused by mechanical abrasion were very sharp. Fillings on the distal surface of the cuspid and the mesial surface of the second bicuspid were broken down by secondary decay, and these surfaces were rough. The patient was very nervous and apprehensive of cancer. He had for some time known of the existence of this growth, but as it gave no particular annoyance for a considerable period after its discovery, he had thought nothing further about it than that it would probably disappear as strangely as it had appeared, if it were not disturbed. But for a month or so previous to my seeing him, he had frequently bitten it while at the table, the result being increased size and tenderness. Careful inquiry concerning his family history gave no evidence of cancerous cachexia. He did not use tobacco in any form.

I first removed the causes of irritation presented by the teeth, by reducing angles to curves and refilling the cavities, the roughness of all the teeth being carefully removed. Then the case was allowed to rest for a few days, for the purpose of studying the effect of this treatment, but not in the expectation that a cure would result from it. The next time I saw it I was gratified to find that it had been subjected to less irritation, the color was better, it was less turgid, and it seemed to be a little reduced in size. It was a very simple matter to remove a tumor located as this one was, and a sufficient amount of healthy tissue was taken up in the removal. The healing of the surface required no attention during the process, except once. The granulations at one point required one application of nitrate of silver. The tumor was immediately placed in Muller's fluid, and subsequently in ninety-five per cent. alcohol, for hardening. This was accomplished in ten days. Sections were then cut, properly stained, and after a suitable immersion in glycerine were mounted in dilute glycerine. (Glycerine seventy-five per cent, distilled water twenty-five per cent.)

It is possible that the interest which centers in the study of disordered epithelium is owing to the fact that very little is known of the many causes of its disease. Certain effects are carefully studied, and from these certain deductions can be safely drawn. We are able to distinguish between a self-explaining tumor and one that apparently has no explanation. The terms benign, dubious, and malignant, have significance, and they are applied by surgeons with intelligence. A careful survey of the subject of epithelioma reveals at once the fact that our knowledge is quite limited. To illustrate: Why should one form of epithelioma in an individual case be properly considered benign, and another form somewhere else in the same subject be as safely considered malignant? Or, to put it another way, if one form of the disease may degenerate to a malignant type, why are not all forms, or any form of epithelioma in a person, whether of a cancerous cachexia or not, likely to degenerate, to soften, to ulcerate, and finally to lead to a fatal result? Will some one explain why one corn on the toe is soft, and another on the same toe hard? The answer might be, that in the hard corn the epidermal cells are more closely packed together in flat layers. In the soft corn the cells are more distinct; they assume the papillary arrangement, and there is more moisture than in the hard corn. But such is not strictly a correct reply, for it is too superficial. The *why* lies deeper. These are the questions of ignorance. In a strictly correct classification a corn or a wart is an epithelioma, as much as a so-called smoker's cancer. The glands of the axilla and groin may swell from very tender warts or corns, but is there a case on record of a malignant ulcer following such effects?

My patient had a hypertrophy of the mucous membrane of his cheek, and he was scared nearly to death—perhaps not without reason—but he was not in the least worried about a wart on his finger, nor a very tender corn on his toe. The only difference was one of locality, and the fact that the derma and cellular fascia beneath the epithelium of the inner cheek is more vascular than it is immediately under the outer epithelium of the body.

Prof. H. H. Smith on surgery says: "The greater vascularity of mucous membrane modifies the formation of epithelioma in this tissue, the induration, or scaly wart, degenerating and ulcerating, and creating, through inflammatory action, more or less induration of the parts immediately adjacent, giving an appearance of depth to the ulcer, which is really quite superficial." But why is it that certain hyper-activity in epithelium will, from some discerned cause, advance into the deeper tissues, unless checked, and develop a malignant form that, by neglect, becomes difficult and occasionally impossible to cure? The difference between a simple hypertrophy and psoriasis is stated by some authorities as consisting in the simple fact that there is no congestive action in a simple hypertrophy, but that in psoriasis there is congestion, heat, the increased action producing squamæ, or scales. The term hypertrophy means excessive nutrition, and whether it be simple or compound, congestion is more or less present and active, but it is not necessarily an inflammation. Now, as to the significance of the terms benign, malignant, etc., applied to morbid growths. Heitzmann says that the term benign is not strictly correct, because a morbid condition is never strictly benign. "Tumors of a so-called benign character may produce distressing and even fatal results by pressure, tension, atrophy of organs, or disturbance of their function." He further says that "The examination of a tumor with the microscope is of the utmost importance, as in many instances it is only by an examination of this kind that a correct diagnosis of the nature of a tumor can be obtained." Again he says: "There are but very few points worthy of consideration as to the nature of a tumor. The more of a basis substance present, the smaller therefore the amount of free bioplasson bodies, the surer it is that the new growth is of a benign nature. On the contrary, the smaller the amount of basis substance, the larger the relative number of free bioplasson bodies, the more certainly does the tumor belong to a malignant type." The distinctions are here drawn with clearness, and I may digress for a moment to say that I think this authority is as clear in fact as in statement, and that the full value of his assertions and claims in his work on the "Morphology of the Animal Body in Health and Disease," will be better appreciated in the future than they now are, and that the spirit of uncharitableness shown him and his views will one day be regretted, to say nothing of mortification at being on record as on the wrong side of *truth.*

The best process of hardening small tumors containing epithelia is by freezing. The freshly removed tumor is carried directly to the microtome, on the drum of which there is placed a thick solution of gum arabic, and in this the specimen is placed and frozen. The sections are quickly carried to the slide, on which they may be stained and subsequently permanently mounted, if desired. But this involves continuous rapid work from the instant of removal from the mouth, and I adopted the process usually followed, and which is referred to in the first part of this paper. In the cutting, the dead epithelia at the apex were lost. Enough, however, in most of the sections was left to show the character of the whole, and the reason of the peculiar color of the apex when viewed *in situ.** The interesting features of this tumor lie beneath these areas of free epithelia, where the changes which occur in the inflammatory pro-

* The drawing, which was done under an amplification of 18 diameters, was made for this paper simply to convey an idea of the general form of the tumor, and fine details would be impossible with such a low power. The engraver's work is, however, very unsatisfactory. The size of the drawing had to be reduced, and instead of correspondingly reducing the details, they are exaggerated.

cess consist, first, in a dissolution or liquefaction of the basis-substance, and secondly, in an increased production of living matter of its own kind. This growth had not passed the stage of tissue formation, and in the centre of the involved territory will be seen a large number of blood vessels; but had it passed to the suppurative stage, the danger of degeneracy would lie in the character of new tissue developed from the parent tissue.

Dr. E. W. Hoeber, of New York, in an article upon Epithelioma in Heitzmann's Morphology, etc., says: "We know that in inflammation the tissue returns to a juvenile condition, and breaks down into elements from which it had sprung. Elements produce their own kind only, when in the embryonal condition. All observers, notwithstanding our limited knowledge as to the cause of the growth of tumors, are agreed that the parent tissue in which a primary new formation originates also returns to a juvenile stage, or stage of indifference, in which it is capable of producing new elements; these, in further development, give rise to the characteristic tissue forms, which are mainly of two kinds: vascularized connective tis-

VERTICAL SECTION OF EPITHELIOMA.

A—Free epithelium. B—Cortical layer of apex. C—Blood vessels. D—Epithelium. E—Juvenile tissue. F—Inflammatory region.

sue, and a vascular epithelium." In the drawing accompanying this paper territories of "free" epithelia and of vascularized epithelia are faithfully shown. Beneath these is shown the tissue in the juvenile stage, or stage of "indifference." This tumor was of slow growth, and a careful examination with high power objectives demonstrates the fact that the epithelia are small. If we remember what Heitzmann has said about certain clinical and microscopical features that constitute a safe guide in distinguishing between the benign and malignant forms of tumors, I think we shall be able, in view of the history and microscopical appearance of this tumor, to decide that it had not yet assumed the malignant form, but that it probably would have done so very soon. Considering its locality, and the certainty of its involving the lymphatics of the region, and very soon after that the parotid gland of that side, the patient is to be congratulated upon what now appears to be a complete cure.

It is not a very long time since an Irish woman, of about sixty years of age, wished me to examine a very loose and somewhat decayed inferior cuspid. The decay was upon the disto-lingual aspect, which was discovered after removal of the tooth, and the subsequent removal of a great quantity of calculi. The tooth was *exceedingly dead*, but all this was of minor importance compared to what I have yet to detail. I noticed an enlargement of the sub-maxillary gland before operating. The swelling extended well down to the clavicle, and the surface of the skin had a peculiar expression, which I attributed to some external poultice, a plan of treatment adopted by the ignorant for relief of toothache. I also noticed a peculiar thickness of speech, that I at first thought was wholly owing to a little of the ardent, and I was excusable in not being very particular in the first examination, because I never before or since stood over such a horrible stench as that which she exhaled with every breath. Midway between this tooth and the point of attachment of the genio-hyo-glossus muscle to the tongue, there was a cone-shaped, dirty, yellow mass, the surface papillary and suppurating at one side. Infiltration had evidently begun. The sub-maxillary, and to some extent the sub-lingual glands, were involved, and the general appearance indicated that exhaustion had already begun. The patient declared that the "lump" under her tongue had been there sixteen years. Possibly it had been there fully as long, but more probably it had not. She stated that her breath had been bad for over a year. There was every indication of a fatal termination in this case, within a few months. The tooth had been a cause of irritation to the tongue at one time, but for years she had not used that side of her mouth in eating, and the accumulated calculi had filled the cavity and covered the crowns of it and the adjoining teeth. But a morbid development had long before commenced. My first thought was Ranula, when I discovered trouble under the tongue after finding such masses of calculi on the teeth, but Wharton's duct was free, and the growth at one side was soon diagnosed as epithelioma.

A thorough rinsing of the mouth with permanganate of potash solution, prepared the patient's mouth for approach and examination. The ulcer was then touched with a solution of chloride of zinc, twenty grains to the ounce of water. After a suitable wash was given the woman, she was sent to her physician, who has since employed constitutional remedies with very indifferent results.

I mention this last case simply to contrast it with the first, and at the same time to show the necessity of early radical treatment of what at first often seems to be a very unimportant morbid condition.

In conclusion, what can be said of the causes of tissual changes from hyper-activities in the mucous membrane, when such an authority as the late Louis Elsberg modestly admitted his ignorance of the subject of inflammation of this tissue?

## Figures and Tables

**Figure f1:**